# Professor Heidenhain’s Views of Mesmerism as Reported by Professor Senator, at the Medical Society at Berlin

**Published:** 1880-09

**Authors:** 


### Selections.

---

### PROFESSOR HEIDENHAIN'S VIEWS OF MESMERISM AS REPORTED BY PROFESSOR SENATOR, AT THE MEDICAL SOCIETY AT BERLIN.

*Gentlemen:*—Willingly yielding to your wish that I should give a report of the experiments upon the hypnotic condition made by Professor Heidenhain in Breslau, I beg that the incompleteness of my report will be excused, as I am not yet fully prepared to make such a report.

I need not remark, that I have regarded these experiments with the greatest skepticism, but I am now convinced that there is no deception, intentional or unintentional. The name of Professor Heidenhain is sufficient guaranty, who has conducted his experiments with the utmost thoroughness, and has kindly allowed me to be a witness with him. The matter investigated is this: Persons by quite simple procedures are thrown into such a condition, that they become, so to speak, involuntary instruments in the hands of those who manipulate them. This procedure consists in causing the object of experiment to gaze fixedly for some time upon a bright object, as a glass knob or the like, and afterwards the operator making passages over the face with the finger-tips so lightly that the skin is scarcely touched, or not at all, or he lays the warm hand upon the forehead; he may also stroke the hair gently, etc.

Many yield to one of these methods, others require that several, one after the other be followed, and for a considerable length of time. Professor Berger, of Breslau, so it is said, has accomplished the same result, simply by bringing near the face a warmed metal plate. All persons are not so susceptible, are not "Mediums." Many, especially after being frequently experimented upon, are so susceptible that they fall into the hypnotic state when looked upon fixedly, or when ordered to fix their attention. This was the condition of the gentleman whom Professor Heidenhain allowed me to examine. They were all physicians or medical students, and simulation by them was not to be thought of.

So far as I have observed, the hypnotic condition began with these symptoms: The individual commenced to breathe deeply and frequently with closed eyes, became at the same time pale, and the eyelids betrayed a trembling movement. In this state the "mediums" imitate what they see or hear. They can easily see, as the eyes are not fully closed; at least they are not always closed, a small opening remains. If the oprator folds the hands, they also fold the hands. Walk in front of them, they imitate the movement, but as though in a deep sleep. When the eyelids are fast closed so that they can not see, they will walk if one steps noisily, being impelled to the motion by the sound only. Similar movement may be made before them and will be likewise imitated.

But what is specially astonishing and capable of throwing some light upon these facts, is in the following observations to which Professor Berger first called attention. A patient whom he had put in the hypnotic state and whom he was about to subject to the electric current, began to speak or to make a sound at the moment he placed the electrode upon the nape and before the current had begun to pass. Heidenhain pursued this discovery and learned something more. Not only when the neck in the region of the upper cervical muscles is pressed upon or stroked, do the hypnotized subjects begin to speak, and, indeed, to repeat what is said to them in a loud tone, but also when other places are thus manipulated, as the epigastrium, apparently at the spot under which the stomach lies in immediate contact with the abdominal wall, and the anterior wall of the larynx. When these places are stroked, and we then speak loudly through a hollow cylinder against them, the subjects mechanically repeat the words. This result will not follow if the experiment be made away from the described regions, even if the change in position be but slight. Heidenhain suspects that these phenomena are due to an irritation of the vagus nerve, which, through reflex action, excites the vocal apparatus. The utterances seem, of themselves, as though the speaker were in a deep slumber, and with great difficulty pronounced the words.

Very remarkable are other observations which remind one of the peculiar reflex phenomena studied by Prof. Goltz. The reaction upon stroking the neck already described, is strongly analogous. But more yet. One subject whom Prof. Heidenhain demonstrated to me, exhibited the following movements when the lateral region of the spine in the lumbar region was stroked; the leg on the same side made scraping movements backwards and could be kept in the position thus attained. Repetition of the stroking caused fresh movements of the same character, and so on until the limb assumed a most unnatural position, and the subject had to be supported lest he should fall. Similar treatment of the other side elicited a similar result. This experiment corresponds probably to the well-known scratching actions in Goltz's dogs.

There was noticed in some that the muscles at the beginning of hypnosis fell into a tetanic rigidity, or into clonic convulsions. In one person these convulsions were so violent, that for fear of general spasms, he was aroused as soon as possible. Recently, it has been discovered that the convulsions can be checked by applying to the extremity affected any cold object. In some very sensitive "mediums" the convulsions cease in both extremities when only one is touched with a cold metal plate.

Prof. Heidenhain finds that among ten persons, three or four only are "mediums." This proportion depends much upon the occupation, mode of life, rank in society. But it is by no means merely the hysterical, nervous and anemic who are thus affected.

The explanation of these phenomena is probably this: The gray matter of the cerebrum (gray matter of the convolutions)

is exhausted by certain fatiguing procedures, and thereby consciousness and the will, as well as the regulation of reflex action are suspended, and as a consequence certain habitual motions, (walking, speaking, writing) take place upon peripheral irritation, sensual impressions, in general, upon excitation that passes inwards to the basal ganglia, and thence avoiding the cortical region, to the periphery again. Naturally through such channels will responses be obtained as are most frequently traversed by impulses, centripetal and centrifugal; hence many will readily exhibit special movements in imitation, which in the case of others will not appear, or at least will be imitated with difficulty.

I have heard that some subjects retain consciousness. In these cases possibly only that part of the cortex is thrown out of activity, which presides over the will.

Prof. Berger, as I am informed, has observed very strange phenomena in the sick; *e. g.*, paretic patients, when hypnotized, moved the affected limb better, which they had previously but little control over. One with marked locomotor ataxia, walked without staggering for a few moments after he awoke from the hypnotism.

The duration of the hypnotism varies sometimes. I saw it last so long that it had to be artificially put to an end, either because the experiment intended was accomplished, or violent convulsions were feared. The subjects were aroused by fanning, calling, striking, shaking, and the like. Occasionally the state was of short duration, and the manipulations had to be repeated and prolonged.

We stand, gentlemen, upon a new field of investigation; one which promises rich results in the department of nervous physiology.—*Berlin. Klin. Woch.*, May 10, '80.

